# One Health approaches to tackling antimicrobial resistance

**DOI:** 10.1016/j.soh.2024.100082

**Published:** 2024-10-21

**Authors:** M.E.J. Woolhouse

**Affiliations:** Usher Institute, University of Edinburgh, Charlotte Auerbach Rd, Edinburgh EH9 3FL, UK

**Keywords:** Antimicrobials, Burden, One Health, Resistance, WASH

## Abstract

Antimicrobial resistance (AMR) is a significant and growing threat to human health. A recent United Nations General Assembly declaration highlights that those in need must have sustained access to effective treatments. In the absence of a reliable supply of new drugs, pressure on existing drugs can be reduced by minimising demand. Routes to reducing demand include: promotion of WASH (access to clean water, sanitation and hygiene) and Universal Health Coverage (UHC); improved infection control in health care settings; and continued efforts to curtail drug use in agriculture. This is a One Health strategy, requiring coordinated action across the human, livestock and environmental sectors.

## 1 Introduction

In September 2024, a United Nations General Assembly (UNGA) High-level Meeting endorsed the text of a declaration on antimicrobial resistance (AMR) [1]. Over one million deaths per year are estimated to be attributable to AMR, and that number is expected to rise without additional interventions [2]. The UNGA declaration should herald a more holistic approach to tackling this threat to global health.

AMR has been described as a quintessential One Health problem [3], having elements of human health, animal health and environmental health. So it is helpful that the UNGA declaration makes multiple references to a One Health approach to tackling AMR.

The declaration also makes reference to the need to use antimicrobials sustainably, given that these drugs continue to save millions of lives. In many parts of the world, lack of access to antimicrobials is a bigger threat to public health than resistance [4], at least for now.

In this perspective, I shall expand on the argument that *“Whatever action we take* [to combat AMR] *will only be sustainable if it is based on a sound understanding of the relative roles of people, animals and the environment in the emergence, spread and persistence of AMR genes”* [3].

## 2 AMR ecosystem

**Fig. 1:**
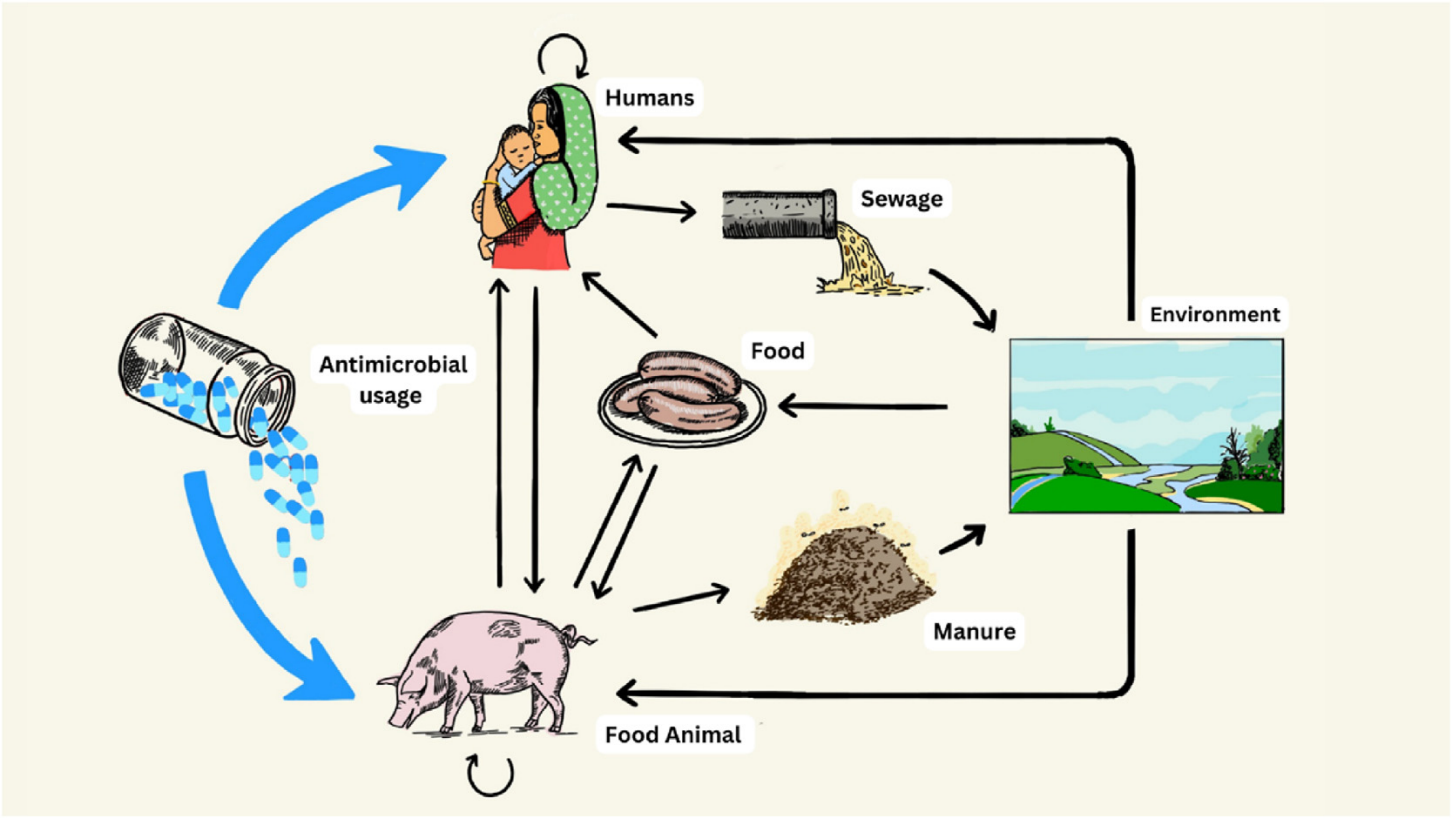
**The antimicrobial resistance ecosystem.** Antimicrobials are used extensively in both humans and food animals (blue arrows). Through movement of the bacteria themselves or mobile genetic elements or drug residues, resistance may spread between humans, food animals and the environment by multiple direct and indirect paths (black arrows). Image credit: Gabrielle Thompson.

Humans, animals and the environment are all part of the AMR ecosystem, connected with one another through direct contact, through food chains, through the use and disposal of manure, through sewage effluent and through pollution (Fig. 1).

A large, observational study in Nairobi, Kenya nicely illustrated this connectivity [5]. By sequencing more than 1000 *Escherichia coli* genomes, it was possible to map the distribution of AMR genes in humans, livestock and wildlife across the city. One useful finding was that livestock keeping, manure management and rubbish disposal all appear to influence the spill-over of AMR genes into wild bird populations [6,7].

The AMR ecosystem is complex and has dynamics driven by non-linearities and feedback loops [8], as can be seen in Fig. 1. This has practical implications because it makes it very difficult to say with confidence what the impact of a potential intervention might be.

For example, we could take steps to reduce the consumption of antimicrobials by food animals. We would expect that intervention to reduce levels of resistance in the animals and their products. But, given that the animals are still part of an interconnected system that includes the production and consumption of antimicrobials for human use, how big might that reduction be?

Or we might take steps to reduce human exposure to antimicrobial resistant bacteria in the environment, including the water supply. How much effect might that have given that humans are also exposed to AMR in settings such as hospitals or through the food chain?

A recent publication has explored both those questions using mathematical models [9]. That study identified a number of scenarios where a greater impact is achieved by tackling the environmental pathway rather than the food animal pathway. These simple models are not intended to provide definitive answers, but they do confirm that these questions need to be asked. If society is to make significant investments in combatting AMR then we need evidence that we have alighted on the most cost-effective interventions.

In a recent report [10], the United Nations Environment Programme (UNEP) also pointed to the paucity of evidence on the functioning of the AMR ecosystem: *“While the relationship between environmental pollution and AMR and the reservoir of resistance genes in the environment has been established, the significance and its contribution to AMR globally is still unclear”.* For example, the high abundance of resistance genes found in wastewater from hospitals can be just a local effect, overwhelmed in sewage systems by the much larger volumes of community wastewater [11]. However, the report went on to state: *“Even so, there is enough knowledge to implement measures to reduce the factors that influence AMR from an environmental perspective”*.

## 3 Surveillance

Another feature of the UNGA declaration is that, for the first time, it proposes global targets, specifically to reduce deaths associated with AMR by 10% by 2030. Having a target requires some means of monitoring progress.

Currently, in many parts of the world, surveillance of the AMR-related health burden remains poor to non-existent [12]. A proposal to add AMR as a possible cause of death to the International Classification of Diseases (ICD) codes used by the World Health Organization [13] has not yet been adopted.

There are also challenges in quantifying levels of resistance. Most current surveillance is based on laboratory culture, an approach that is time-consuming and difficult to scale up. An alternative is to carry out metagenomic analysis of wastewater samples to quantify the abundance of AMR resistance genes. This approach has several advantages: it covers large whole communities, not just patients; it is relatively cheap, flexible, scalable and quick to implement; and it is easily standardised [14]. Wastewater sampling is not a replacement for clinical surveillance – they perform different roles – but it has considerable potential as a monitoring tool, particularly in countries with limited capacity in laboratory microbiology.

For example, a comparison of AMR gene abundance in wastewater across 79 sites globally revealed high levels of resistance in Africa [15], despite antimicrobial consumption in the region being comparatively low. More recent work has shown that, based on wastewater sampling, the repertoire of AMR genes – the resistome – in Africa is quite distinct from that in Europe and North America [16].

Statistical analysis of these data has shown that, on a global scale, high AMR gene abundance in wastewater is more strongly associated with poor sanitation and weak health systems than with antibiotic usage [17]. The implication of this body of work is that high levels of AMR in Africa and elsewhere are being driven by high rates of bacterial transmission, resulting in a relatively high public health burden due to AMR even in populations with below average access to antibiotics [18].

## 4 Water, sanitation and hygiene

Access to clean water, sanitation and hygiene (WASH) has been recognised as a human right since 2010. Yet, according to a recent report [19], despite recent progress, over two billion people worldwide still lack access to safe drinking water, half the global population does not have access to safe sanitation, three billion people do not have access to handwashing facilities with soap, and over 400 million people still practice open defecation.

A recent study [2] points out that a decline in AMR mortality in children under five years since 1990 coincides with steady improvement in access to WASH, and forecasts these trends to continue. Others have linked AMR to the broader issue of Universal Health Coverage (UHC), which encompasses treatment as well as prevention [20]. Continued progress towards WASH for all and UHC should further limit the health burden of AMR.

## 5 Antimicrobials in food animals

Globally, antimicrobial usage in food animal production has overtaken usage in human medicine and continues to grow as livestock production becomes more intensive [3]. The absolute volume of consumption is highest, and is still growing, in China and Brazil.

There is an active debate about the significance of these levels of consumption for human health. In summary, although scientific opinion is largely in favour of reducing livestock consumption of antimicrobials [21], direct evidence of the human health benefits of doing so is harder to find, except for people working in direct contact with livestock [22].

Again, simple mathematical models can provide useful insights into the problem [23]. Due to the interconnections between components of the AMR ecosystem in Fig. 1, interventions targeted solely at livestock may not have a substantial impact on AMR in humans. The same applies in reverse. In other words, reducing usage of antimicrobials in livestock is necessary but not sufficient. This answer fully aligns with a One Health approach: the greatest impact is expected when there is a coordinated effort to reduce consumption by both livestock and humans.

This proposition is proving much harder to demonstrate in practice, but statistical analysis of spatiotemporal data from Europe has found cross-correlations between usage in food animals and resistance in humans and vice versa [24].

## 6 Sustainability

The 2024 UNGA declaration, reflecting the evidence set out in a recent *Lancet* series on AMR [25], puts more emphasis on access to and sustainable use of antimicrobials. This is a noticeable shift from previous work on this topic that stressed the need to reinvigorate the drug discovery and development pipeline [21]. Both are important, but while increasing the supply of new antimicrobials is a long-term aspiration, reducing the demand for antimicrobials across the board is more immediately achievable.

As discussed above, WASH and UHC reduce the burden of bacterial infections, thereby reducing demand for antimicrobials. There is already evidence that the impact of lower levels of bacterial-related disease is outweighing increasing levels of resistance, particularly among children (though with the important exception of sepsis) [2]. Alongside the many benefits of WASH, it is also a comparatively cheap intervention. WASH for all would cost an estimated US$114 billion per year [26], whereas the economic cost of AMR is set to exceed US$1 trillion per year by 2030 [27].

Beyond WASH, more could be done to improve infection prevention and control in healthcare settings, as well as better stewardship, such as clamping down on inappropriate and unnecessary use. These will become even more important in the next two decades as the AMR burden is expected to shift markedly to the older members of the population who are especially vulnerable to health-care associated infections [2].

Also, more could be done to reduce the usage of antimicrobials in food animal production. Although some countries, and some sectors, have had some success in this direction [28], overall, any progress has been outweighed by the growth of the industry globally, fuelled by increasing demand for animal food products as living standards rise [29].

Antimicrobials remain “an essential part of modern life” [10], now and for the foreseeable future, so we have to find ways of using them sustainably. That grand vision is compromised by a lack of understanding of what sustainable use looks like in practice. It remains unclear whether demand can be reduced to the point that levels of resistance stabilise, nor even where that point might be. These are the kinds of questions that scientific research can answer.

Science has played and will continue to play a key role in formulating national and global AMR strategies. Recognising this, a further recommendation of the UNGA declaration is to set up an independent panel that would facilitate the generation and use of multisectoral scientific evidence in tackling AMR. That panel will have important and urgent work to do; coming up with a clear vision for sustainable antibiotic use should be right at the top of the list.

## CRediT authorship contribution statement

**M.E.J. Woolhouse:** Writing – original draft.

## Funding

This work was supported by grants from the 10.13039/501100009708Novo Nordisk Foundation (grant number NNF16OC0021856: Global Surveillance of Antimicrobial Resistance) and the 10.13039/501100000780European Commission (grant number 874735: VEO).

## Declaration of competing interest

The authors declare the following financial interests/personal relationships which may be considered as potential competing interests: M.E.J. Woolhouse reports financial support was provided by European Commission. M.E.J. Woolhouse is a Guest Editor for an special issue (Rethinking Public Health Paradigms: systems thinking with One Health approach) of *Science in One Health*.
